# Supplementary figures and images for: Increasing methane (CH4) emissions and altering rhizosphere microbial diversity in paddy soil by combining Chinese milk vetch and rice straw
Source: PeerJ. 2020 Aug 3;8:e9653. doi: 10.7717/peerj.9653 (PMC7409806; doi:10.7717/peerj.9653)

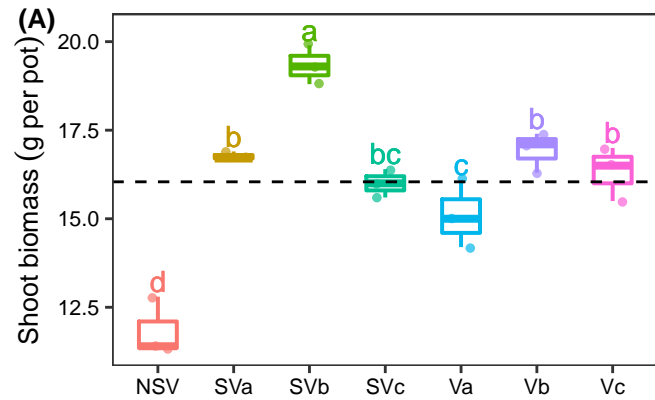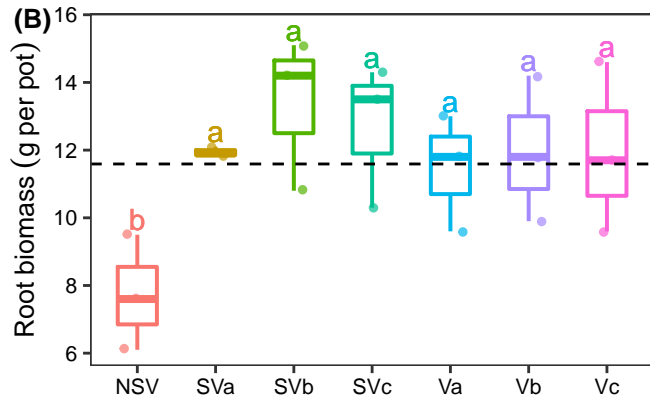

Supplement: Supplemental Information 1 — S, straw; V, vetch; a = 0, b = 15, c = 30 kg ha−1 N from vetch; NSV, no straw and vetch. Different letters represent significant differences (P < 0.05) (n = 3). [file peerj-08-9653-s001.pdf]

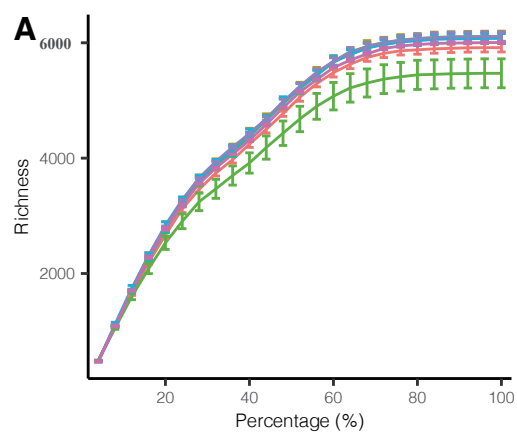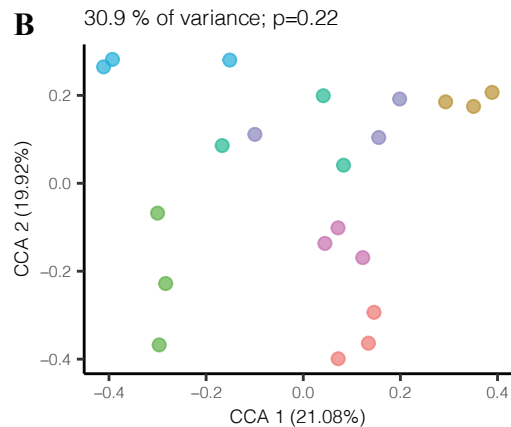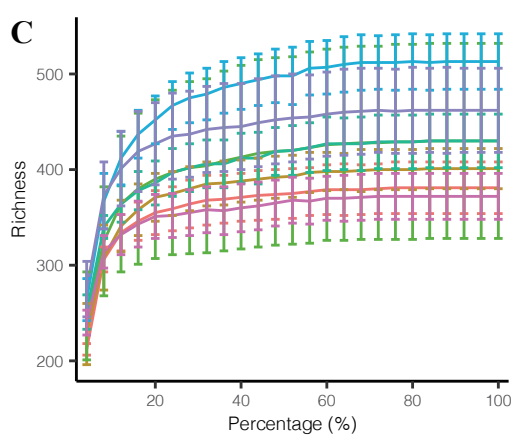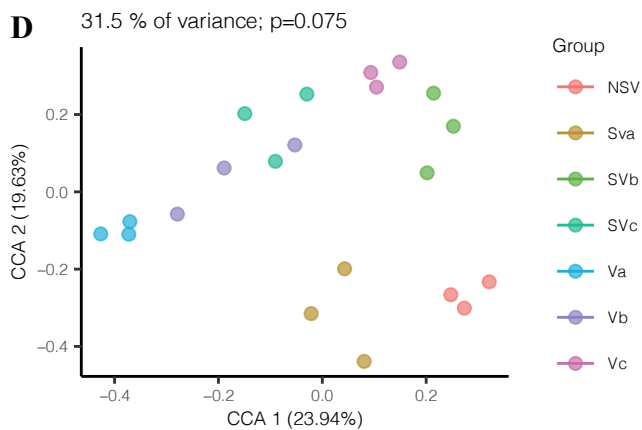

Supplement: Supplemental Information 2 — (A) Bacterial 16s rDNA gene rarefaction curves for the different treatments, with standard errors. (B) Fungal ITS1 gene rarefaction curves for the different treatments, with standard errors. (C) 16s rDNA gene constrained CCA. (D) ITS1 gene constrained CCA. [file peerj-08-9653-s002.pdf]
