# Supplementary material for: Increasing methane (CH4) emissions and altering rhizosphere microbial diversity in paddy soil by combining Chinese milk vetch and rice straw
Source: PeerJ. 2020 Aug 3;8:e9653. doi: 10.7717/peerj.9653 (PMC7409806; doi:10.7717/peerj.9653)
Supplement: Supplemental Information 4 — S, straw; V, vetch; a = 0, b = 15, c = 30 kg ha−1 N from vetch; NSV, no straw and vetch. [file peerj-08-9653-s004.pdf]

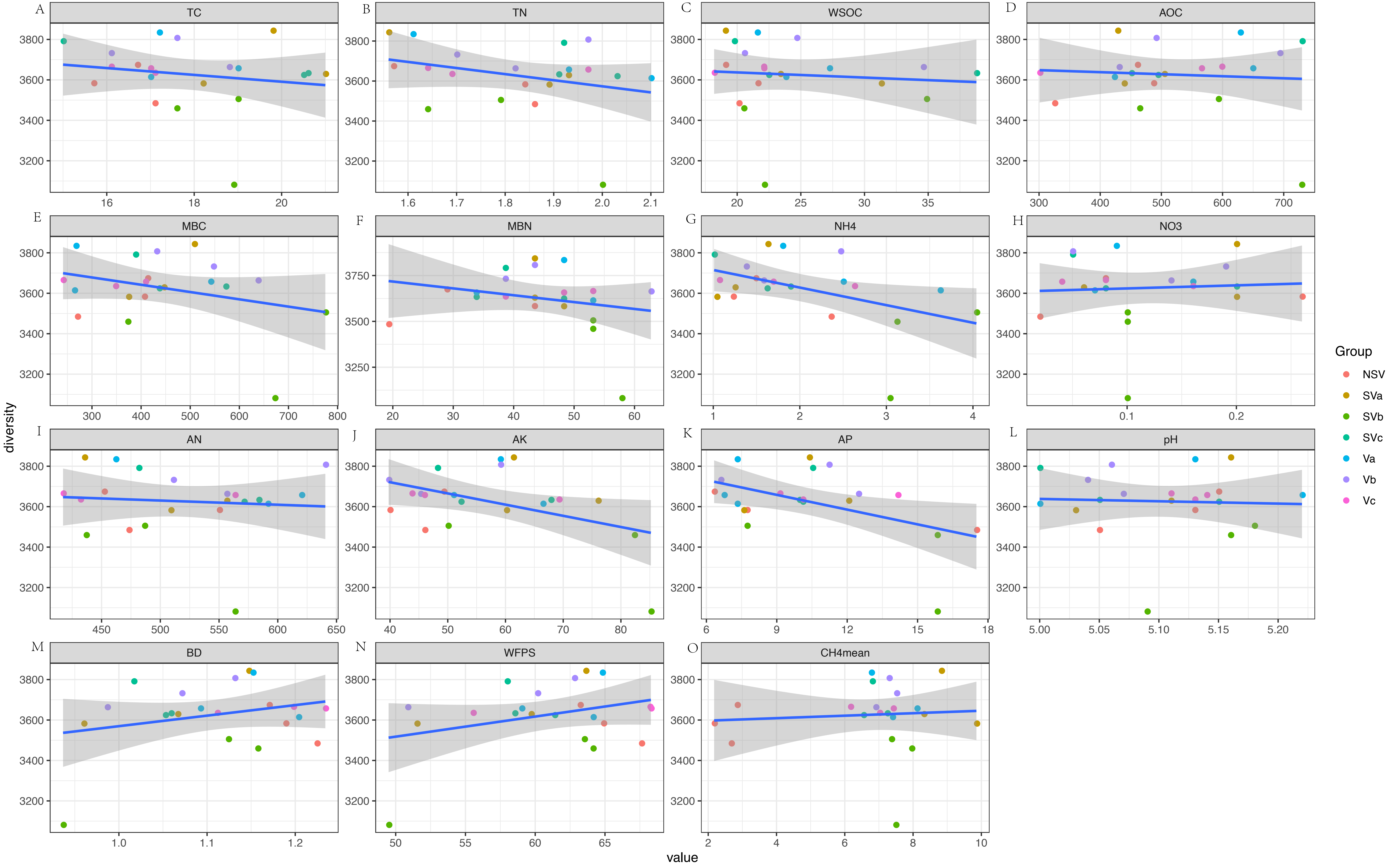

S Fig. 1 The relations between baterial community and environmental factors. S, straw; V, vetch; a = 0, b = 15, c = 30 kg ha−1 N from vetch; NSV, no straw or vetch. TC, total carbon; TN, total nitrogen; AOC, activated organic carbon; MBC, microbial biomass carbon; MBN, microbial biomass nitrogen; NH4-N, ammonium nitrogen; NO3-N, nitrate nitrogen; AN, alkali-hydrolysable nitrogen; AK, available potassium; AP, available phosphorus; BD, bulk density; WFPS, water-filled pore space; CH4mean, cumulative emission of methane in the early rice season(from pot experiment); .
